# Carotenoids from Marine Sources as a New Approach in Neuroplasticity Enhancement

**DOI:** 10.3390/ijms23041990

**Published:** 2022-02-11

**Authors:** Sylwia Pietrasik, Natalia Cichon, Michal Bijak, Leslaw Gorniak, Joanna Saluk-Bijak

**Affiliations:** 1 Department of General Biochemistry, Faculty of Biology and Environmental Protection, University of Lodz, Pomorska 141/143, 90-236 Lodz, Poland; sylwia.pietrasik@edu.uni.lodz.pl (S.P.); joanna.saluk@biol.uni.lodz.pl (J.S.-B.); 2 Biohazard Prevention Centre, Faculty of Biology and Environmental Protection, University of Lodz, Pomorska 141/143, 90-236 Lodz, Poland; michal.bijak@biol.uni.lodz.pl (M.B.); leslaw.gorniak@biol.uni.lodz.pl (L.G.)

**Keywords:** marine carotenoids, neuroplasticity enhancement, fucoxanthin, astaxanthin, mytiloxanthin, siphonaxanthin, saproxanthin, myxol

## Abstract

An increasing number of people experience disorders related to the central nervous system (CNS). Thus, new forms of therapy, which may be helpful in repairing processes’ enhancement and restoring declined brain functions, are constantly being sought. One of the most relevant physiological processes occurring in the brain for its entire life is neuroplasticity. It has tremendous significance concerning CNS disorders since neurological recovery mainly depends on restoring its structural and functional organization. The main factors contributing to nerve tissue damage are oxidative stress and inflammation. Hence, marine carotenoids, abundantly occurring in the aquatic environment, being potent antioxidant compounds, may play a pivotal role in nerve cell protection. Furthermore, recent results revealed another valuable characteristic of these compounds in CNS therapy. By inhibiting oxidative stress and neuroinflammation, carotenoids promote synaptogenesis and neurogenesis, consequently presenting neuroprotective activity. Therefore, this paper focuses on the carotenoids obtained from marine sources and their impact on neuroplasticity enhancement.

## 1. Introduction

In the past few decades, the quality of human life has significantly improved due to advances in medicine, lifestyle and nutrition changes. At the same time, it contributed to the augmented lifespan and, therefore, the growing number of the elderly, in whom, with age, ailments from miscellaneous central nervous system (CNS) diseases occur. In this regard, scientists have started to put more emphasis on being better acquainted with the processes leading to nervous tissue pathology, which would allow them to develop effective drugs to improve neurological recovery. The studies on carotenoids with potent antioxidant and anti-inflammatory power, which could be possible applications in neuro-intervention, have been conducted for quite some time. Furthermore, recent results have indicated they also own neuroprotective activity and stimulate synaptogenesis and neurogenesis, by, for instance, inhibiting oxidative stress and neuroinflammation, which makes them important compounds in neuroplasticity enhancement and CNS therapy [1].

The nervous system’s capacity to undergo maturation, modify its structure and function, adapting to both physiological and pathological variations in the environment, is known as neuroplasticity. Without this ability, any brain would be unable to develop from infancy through to adulthood or recover from injury [2]. It is a complex physiological process, characterized by a limited scope, happening in the brain for its whole life. Therefore, it comprises neurogenesis, synaptogenesis, and neurochemical variations of the CNS. Brain plasticity is realized mainly by modulating genetic, molecular and cellular mechanisms that influence synaptic connections and neural circuitry formation [3]. High clinical hopes in regulating neuroplasticity processes are raised by both pharmacotherapies and biological therapies, which neurorestorative activity is realized through a synergistic effect occurring between neurogenesis and synaptogenesis.

Neurogenesis, regulated by a wide range of factors, including neurotrophins, neurotransmitters and growth factors, is one of the components of brain plasticity. Neurons, in that process, are generated from neural stem cells (NSCs) and integrated into existing neuronal circuits [4]. NSCs are required for the adequate functioning of neurogenesis by retaining their self-renew moldability and generating neuronal precursors throughout life. In the adult brain, neurogenesis is mainly localized in the subventricular zone (SVZ) and the subgranular zone (SGZ), which are responsible for memory, learning, and olfactory sensation [5]. However, with age, the ability of the newly formed nerve cells to survive as well as the rate of neurogenesis decreases. Adulthood neurogenesis perturbation contributes to various human disorders, such as cognitive impairment or neurodegenerative diseases [6]. In turn, synaptogenesis, which creates new neural connections, occurs throughout life. In adults, synaptogenesis remains a local event, founded on creating new connections and improving existing synaptic pathways [7].

Growth factors, including brain-derived neurotrophic factor (BDNF), nerve growth factor (NGF), and fibroblast growth factor (FGF), play prominent roles in modulating brain plasticity by activating signaling pathways. Examples include: phosphoinositide-3-kinase–protein kinase B/protein kinase B (PI3K/Akt), mitogen-activated protein kinase/extracellular signal-regulated kinase 1/2 (MAPK/Erk), and phospholipase C/inositol trisphosphate/Ca2+/calmodulin-dependent protein kinase II (PLC/IP3/CAMKII), involved in neuron proliferation and survival as well as neuroprotection [8,9,10,11]. Mature forms of neurotrophin bind to a member of the tyrosine kinase receptor family, the tropomyosin receptor kinase (Trk) and to a representative of the tumor necrosis factor receptor superfamily, p75 receptor. They regulate survival, proper development, normal neuronal function, and synaptic strength and plasticity through them [12,13]. Trk receptors (TrkA, TrkB and TrkC) are composed of ligand-binding domains, the transmembrane domain and the cytoplasmic domain. Those domains contain several sites of tyrosine phosphorylation that recruit intermediates in intracellular signaling cascades [14]. The direct proteins binding to Trk receptors leads to tyrosine kinases activation and, in consequence, activates several proteins, including Ras, Ras-related protein 1 (Rap-1), as well as pathways regulated by MAPK, PI3K, and PLC-γ [15]. Unlike the Trk receptors, which autophosphorylate after ligand engagement, the p75 receptor does not contain a catalytic domain to autoactivate. Therefore, it functions mainly via interactions with other effector proteins, mainly by signaling, promoted by the Trk receptors and modulating their functions [16]. Ras proteins activate the PI3K/Akt pathway causing the activation of the expression of genes involved in brain plasticity or MAPK/Erk pathway. That leads to the transcription of protein factors engaged in neurogenesis and synaptogenesis, including cAMP response element-binding protein (CREB), Myc, and ribosomal S6 kinase (RSK) [17].

PI3K activation, stimulated by Ras, is a critical signaling pathway responsible for neurons survival [18]. PI3K generates phosphatidyl inositides accountable for activating protein kinase Akt, also termed protein kinase B (PKB). It has an influence on many proteins involved in regulating cell survival. For instance, PKB inhibits apoptosis by Bcl2-associated agonist of cell death (BAD) phosphorylation [19]. Akt also influences the nuclear factor kappa B (NFκB) pathway. After stimulation with specific or unspecific signals (oxidative stress, inflammatory cytokines), nuclear factor kappa B inhibitor (IκB) phosphorylated by the IKK complex leads to the ubiquitination and proteasomal degradation of the IκB protein and the NFκB stimulation. That mechanism activates the transcription of various target genes, many of which are inflammatory and immunoregulatory, which modulates the neurons’ survival [20].

Akt is associated with both inhibition and promotion of apoptosis, by phosphorylating the transcription factor forkhead 1 (FKHRL1), which regulates apoptosis-promoting proteins expression, and by the negative regulation of glycogen synthase 3β kinase (GSK-3β), respectively. Furthermore, PI3K signaling may also be initiated in the Ras-independent manner when PI3 kinase binds to the growth factor receptor-bound protein 2 (Grb-2)-associated-binding protein 1 (Gab-1) activated by phosphorylated Grb-2 [15,21].

MAPK/Erk pathway, induced by Ras, is activated by the Src homology and containing protein (Shc)/Grb-2/son of sevenless (SOS). Besides, it can also be stimulated by Trk, which phosphorylates fibroblast growth factor receptor substrate 2 (FRS-2), causing its binding to the adapter molecule crk, which associates with the guanyl-nucleotide exchange factor (C3G), that in turn stimulates Rap1. Protein Rap1 activates the Erk kinase signaling pathway. The Erk kinase, through the RSK and MAP pathway, phosphorylates CREB and other transcription factors, regulating genes expression responsible, among others, for the neurons’ survival [15]. In addition, the Trk receptor phosphorylation leads to the PLC-γ1 activation, causing diacylglycerol (DAG) and IP3 formation [22]. IP3 induces Ca2+ reservoirs release and thus increases its cytoplasmic level, indirectly prompting the action of many enzymes, including CAMK and calcium-modulated protein (calmodulin)-dependent phosphatase [23]. In contrast, DAG stimulates DAG-dependent protein kinase C isoforms (PKCδ) activity, which induces the MAPK/Erk pathway [24].

Since multiple brain processes are affected by natural substances like carotenoids, including neurogenesis, synaptic plasticity, and neuronal connectivity, the therapy, based on these compounds, seems to be a promising treatment strategy for CNS diseases. Furthermore, several carotenoids, generously present in marine organisms and easily digestible, exhibit positive effects on brain function enhancement. Therefore, this work aims to review the latest research on the use of carotenoids from marine sources in neuroplasticity enhancement.

## 2. Neurorestorative Actions of Marine Carotenoids

**Figure 1:**
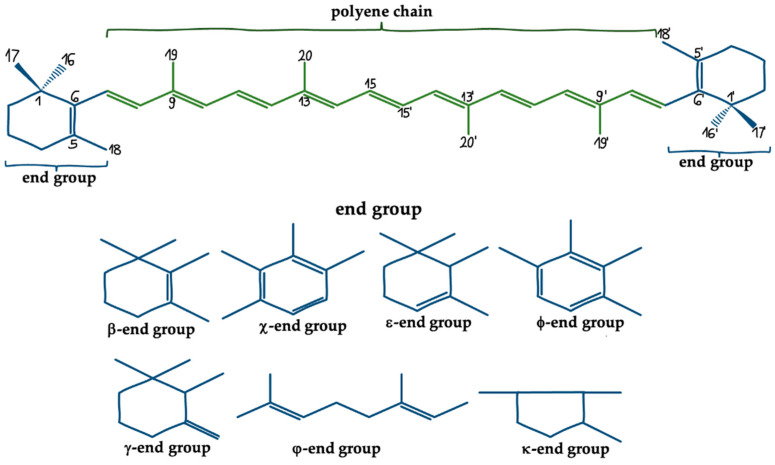
The chemical structure of carotenoids and their end groups.

Basically, carotenoids consist of a polyisoprenoid structure, a long-conjugated chain of double bond and an end group at both ends of the chain [25]. They can be categorized into carotenes, containing a hydrocarbon chain and xanthophylls, oxygen derivatives of carotenes, forming hydroxyl, epoxide, and keto groups [26]. The functions of carotenoids from aquatic habitats are largely specified by their molecular properties such as size, geometry, functional groups, and other traits [25] Kliknij lub naciśnij tutaj, aby wprowadzić tekst. Most of them are lipophilic and can cross the blood-brain barrier (BBB), which is fundamental during neuroplasticity enhancement, treatment of brain injuries or the prevention of brain disorders with these molecules [27,28]. The typical structure of carotenoids and different end groups are shown in Figure 1.

In humans, carotenoids play different significant functions in the brain and have several medicinal properties, including neuroplasticity enhancement [29,30,31,32]. They wield essential roles in immunity, take part in the antioxidant defense system, improve brain function, and are linked to a reduced risk of acquiring chronic diseases. The pharmacological properties, such as antioxidant, anti-inflammatory, and anti-apoptotic potentials of marine carotenoids, endorse their protecting effectiveness against oxidative stress, neuroinflammation and mitochondrial dysfunction, which are known to be implicated in brain injuries or neurodegenerative diseases pathophysiology [33]. Marine carotenoids are suggested to impact gene expression and cell function through multiple mechanisms, especially by: interacting with several transcription factors, including BDNF, NGF, NFκB; modulating signaling pathways, such as the NFκB, MAPK, and the nuclear factor erythroid 2-related factor 2 (Nrf2), associated with inflammatory and oxidative stress responses; and scavenging of reactive oxygen species (ROS) [34]. Hence, these compounds can act either directly on biological molecules and systems or indirectly through the expression of different genes engaged in, among others, antioxidant responses.

Due to their conjugated double-bond structure, carotenoids from the aquatic environment are strong scavengers of singlet oxygen and peroxyl radicals. They act as chemical quenchers of singlet oxygen. Three major types of reactions of free radical scavenging by carotenoids are: electron transfer between the free radical and carotenoid, whereby a carotenoid radical cation or carotenoid radical anion is formed; radical adduct formation; hydrogen atom relocation leading to a neutral carotenoid radical [35].

In addition to their scavenging function toward ROS, carotenoids may also operate through more indirect tracks, including Nrf2, NFκB, or MAPK signaling pathways [36,37].

The Keap1-Nrf2 pathway plays a vital role in the cellular defense against ROS. Moreover, Nrf2 signaling is an important molecular mechanism for neuroprotection and it modulates the activation of immune cells, including microglia. Under normal conditions, Keap1 promotes ubiquitination and degradation of Nrf2, thus maintaining it in an inactive form in the cytosol. During redox imbalance, the Keap1-Nrf2 association is disrupted, Nrf2 ubiquitination is inhibited, leading to its accumulation in the cell and translocation to the nucleus. There, it binds to the antioxidant response element (ARE), leading to antioxidant and cytoprotective enzymes expression. Marine carotenoids interact with Keap1 by changing its conformation, resulting in enhancement of antioxidant activity [38,39,40].

NFκB is accountable for the transcription of various genes that regulate inflammatory responses. Under resting conditions, NFκB is bound to IκB, which resides in the cytoplasm. However, during chronic neuroinflammation, carotenoids or their derivatives may block NFκB activation by interaction with cysteine residues of the IKK and/or NFκB subunits. That NFκB activation blocking causes the target genes transcription repression and thus diminishes inflammation and increases neurons survival [41].

In addition to the NFκB pathway, the anti-inflammatory effects of marine carotenoids are also found through regulating other pathways, including Akt and MAPK pathways, which control synaptic plasticity in the adult brain. Carotenoids from aquatic habitats may increase the phosphorylation of Akt, and phosphorylated Akt regulates Nrf2 and NFκB, influencing gene expression [42,43]. Thereby it alleviates oxidative stress or inflammation-associated damage in brain cells [44]. In the MAPK/Erk pathway, phosphorylated Erk translocates into the nucleus where it activates transcription factors such as Elk-1 and Msk. That activation of the transcription factors regulates synaptic plasticity and, consequently, it may contribute to the neuroplasticity enhancement [45,46].

Besides, marine carotenoids may impact the level of several neurotrophic factors, including NGF or BDNF. BDNF, the major neurotrophin in the brain, being able to activate intracellular signaling via binding to its receptors, is critical in the proper functioning of the nervous system because it regulates neuronal survival and differentiation, learning and memory. Additionally, BDNF plays a role in proteins’ transcription and translation, which are engaged in synapse development. BDNF is also an underlying agent in the pathology of neural diseases like Alzheimer’s disease, schizophrenia and depression [47,48]. In turn, NGF plays a crucial role in developmental and adult neurobiology for its significant regulatory activity at nerve cells survival, growth, and differentiation in the CNS [49]. Carotenoid supplementation might increase systemic levels of BDNF reduced during neuroinflammation, and the plausible mechanism for this effect is marine carotenoids anti-inflammatory capability [50,51,52]. They also promote the secretion of NGF and BDNF from NSCs [42]. BDNF can block neuronal apoptosis by inducing phosphorylation of Akt during excitotoxicity [53]. In contrast, NGF regulates apoptosis by activating PI3K/Akt and MAPK/Erk pathways [54]. NGF may interact with TrkA and activate Erk signaling to phosphorylate some proteins, as Bcl-2-like protein 11, and inactivate their pro-apoptotic function [55].

Carotenoids derived mainly from marine sources, such as astaxanthin (AST), fucoxanthin (FUC), but also the rare siphonaxanthin or myxol, have lately shown antioxidant and inflammatory effects, which help enhance cognitive function and neuroprotection. Since carotenoids are hydrophobic antioxidants, their main action mechanism is found within biological membranes and depends on their structural features and membrane composition. The results showed that AST, having two polar hydroxyl groups, is anchored across the membranes with polar functional groups oriented outside. Hence it exhibits more effective protection against oxidation by peroxyl radicals than β-carotene or lutein, which are oriented parallel to the membrane surface [56]. Recent reports claim that AST delays or ameliorates cognitive impairment associated with normal ageing or alleviates various neurodegenerative diseases’ pathophysiology [57,58]. AST proved its neuroprotective potential by preventing brain damage in progeny exposed to prenatal epilepsy seizures by inducing the expression of CREB and BDNF in the hippocampus of newborn rats [59]. Additionally, another typical marine carotenoid—FUC reduced Aβ-induced damage in a cultured cell model through apoptotic factors downregulation, inflammatory cytokine-mediating action inhibition, and simultaneous ROS reduction [60].

**Figure 2:**
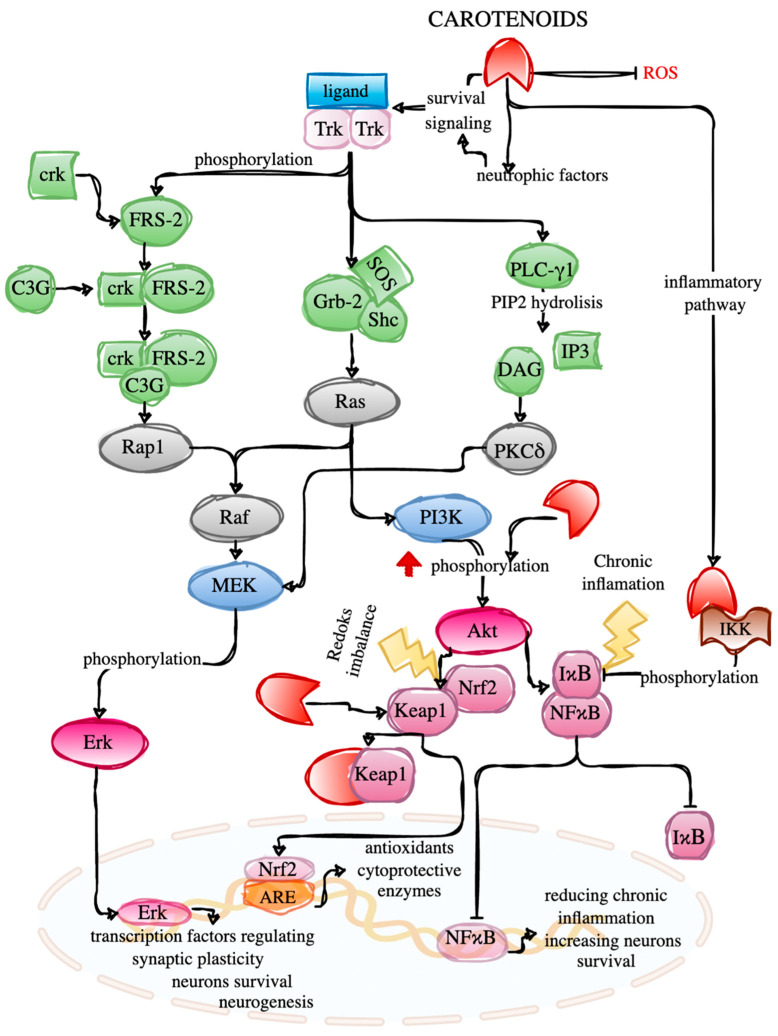
Marine carotenoids actions on signaling pathways enhancing neuroplasticity. Abbreviations: Akt—protein kinase B; ARE—antioxidant response element; crk—CT10 regulator of kinase; C3G—guanyl-nucleotide exchange factor; DAG—diacylglycerol; Erk—extracellular signal-regulated kinase; FRS-2—fibroblast growth factor receptor substrate 2; Grb-2—growth factor receptor-bound protein 2; IκB—inhibitor of NFκB; IKK—IκB kinase; IP3—inositol trisphosphate; Keap1—kelch-like-ECH-associated protein 1; MAPK—mitogen-activated protein kinase; MEK—mitogen-activated protein kinase kinase; NFκB—nuclear factor kappa B; Nrf2—nuclear factor erythroid 2-related factor 2; PI3K—phosphoinositide-3-kinase–protein kinase B; PKC—protein kinase C; PLC—phospholipase C; Raf—rapidly accelerated fibrosarcoma; Rap1—Ras-related protein 1; ROS—Reactive oxygen species; Shc—Src homology and containing protein; SOS—son of sevenless; Trk—tropomyosin receptor kinase.

Besides, other carotenoids also positively impact CNS recovery. High levels of lutein and zeaxanthin within the brain can improve cognitive function in the elderly by their neuroprotection ability to neuronal mortality reduction [61]. Additionally, β-carotene exhibited its potential in the treatment of acute spinal cord injury, the inhibition of the NFκB pathway reduced the progression of secondary injury events [62]. Moreover, lycopene proved to improve neurological function recovery by suppressing neuronal death and neuroinflammation in spinal cord ischemia/reperfusion injury rat models [63]. The schematic depiction of marine carotenoids actions on signaling pathways related to neuroplasticity is presented in Figure 2.

Increased ROS causes oxidative stress inside cells. Marine carotenoids can lower ROS level, thereby mitigating cellular damage and inhibiting inflammatory responses as well as participating in the maintenance of neuronal plasticity. In the MAPK/Erk pathway, phosphorylated Erk translocates into the nucleus where it activates transcription factors responsible for synaptic plasticity regulation. Certain carotenoids from aquatic ecosystems increase the phosphorylation of Akt, and phosphorylated Akt regulates Nrf2 and NFκB, influencing gene expression. Thereby it alleviates oxidative stress or inflammation associated damage in brain cells. Under natural conditions, Nrf2 is kept inactive by its repressor protein Keap1 in the cytosol. During redox imbalance, the Keap1-Nrf2 linkage is disrupted, and marine carotenoids seem to change Keap1 conformation, resulting in Nrf2 liberation and translocation to the nucleus. There, it binds to the ARE, causing antioxidant and cytoprotective enzymes expression. Under resting conditions, NFκB is bound to IκB (inhibitors of NFκB), which resides in the cytoplasm. However, during exposure to specific or unspecific signals (oxidative stress, inflammatory cytokines), the IKK complex phosphorylates IκB protein which leads to its ubiquitination and proteasomal degradation and the NFκB pathway activation. NFκB translocates into the nucleus, where it could bind to DNA sequences, activating the transcription of various target genes, many of which are inflammatory and immunoregulatory. During chronic neuroinflammation, blocking NFκB activation, for instance, by some marine carotenoids, will lead to the transcription of the target genes repression and thus reduce inflammation and increase neurons survival. In addition, these carotenoids may impact the level of several neurotrophic factors which can regulate survival signaling pathways.

Importantly, it has been shown that marine carotenoids may modulate autophagy, which is associated with the neutralization of damaged organelles, including misfolded proteins in the CNS [64]. Some carotenoids, depending on the conditions and the type of research material, showed a different regulating potential of autophagy. Lutein in rat Müller cells inhibited autophagy through the mTOR pathway [65], while in retinal pigment epithelial cells it activated autophagy through Beclin-1 overexpression [66]. Similarly, crocin, depending on the amount of oxygen, influenced autophagy in two ways: hypoxia activated it, while reperfusion extinguished it [67]. The autophagy-inhibiting potential is also shown by astaxanthin [68] and lycopene [69], while FUC demonstrates the autophagy-promoting activity [70]. This inconclusive evidence of carotenoids modulating autophagy implies further research into the phenomenon. This seems to be particularly important in relation to supporting the management of neurodegenerative diseases.

## 3. Marine Carotenoids

Marine and oceanic fauna and flora represent an enormous wealth of potential therapeutic agents, and amongst them, over 250 nautical carotenoids, which, generally, serve as natural, lipid-soluble pigments responsible for nature’s varied and vivid colors [71]. Humans cannot synthesize carotenoids de novo, therefore they must be obtained through the diet and converted into functional metabolites. Moreover, considering their low bioavailability in humans, different strategies, for instance, encapsulation in liposomes, micelles, or nanogels, increasing their absorption efficiency in the digestive tract have been developed [72,73]. Bioactive metabolites of marine algae, fungi, diatoms, and other marine organisms have been identified as pharmaceuticals with a wide variety of uses [71]. The significant role of marine carotenoids in neuroplasticity is underlined by the fact that there are currently several clinical studies conducted on the effects of carotenoids on cognitive impairment, neuroprotection, oxidative stress, and neurodegenerative diseases [74,75,76].

### 3.1. Fucoxanthin

One of the most promising carotenoids to be used in CNS diseases is FUC, the source of which is brown algae, mainly *Sargassum siliquastrum*, *Undaria pinnatifida*, *Hijikia fusiformis*, *Alaria crassifolia*, *Laminaria japonica,* and *Cladosiphon okamuranus* [77]. FUC is a naturally occurring compound with a brown color, showing the activity of provitamin A. This xanthophyll contains in its structure an epoxy group and conjugated carbonyl groups in the polyene chain. This structure translates into the antioxidant properties of FUC [78,79]. The biological activity of FUC is associated with a strong anti-inflammatory [80,81], antioxidant [82], anticancer and cell cycle suppressing [83,84], antidiabetic [85], hepatoprotective [86] and cardioprotective effects [87].

The neuroprotective effect of FUC has been confirmed in several in vitro and preclinical studies. It is proposed that Nrf2 signaling is the most important molecular mechanism for neuroprotection in FUC [88,89,90]. Hu et al. [88] evaluated the neurorestorative properties of FUC in a rat stroke model. The animals were administered this carotenoid at a dose of 30, 60, and 90 mg/kg 1 h prior to ischemia induction, and then rat cortical neurons were harvested and treated with 5, 10, and 20 µM FUC. It was observed that FUC dose-dependently reduced neurological deficits and infarct volume. Moreover, FUC blocked apoptosis by reducing the elevated cleaved caspase (C-CASP) 3 and Bcl-2/Bax ratio and also decreased oxidative stress by increasing SOD activity. The neuroprotective properties of FUC were confirmed in an in vitro study where a dose-dependency reduction of ROS accumulation and apoptosis was demonstrated through activating the Nrf2/HO-1 pathway initiated by Nrf2 nuclear translocation and increased levels of HO-1 [88]. Wu et al. [89] also suggested an impact of FUC on the activation of Nrf2 signaling, albeit by inhibiting the interaction of the Keap1 repressor protein with Nrf2. In 6-hydroxydopamine (6-OHDA) induced PC12 cells, FUC reduced ROS accumulation, cell apoptosis and membrane potential interference, as well as dose-dependently enhanced the activity of antioxidant enzymes: glutamate-cysteine ligase modifier subunit, nicotinamide heme oxygenase-1, and glutamate-cysteine ligase catalytic subunit. The FUC was then administered to the zebrafish treated with 6-OHDA. It was observed that FUC improved the granular region of the brain injury and enhanced the total swimming distance of the larvae [89]. Activation of the Nrf2/ARE pathway was also confirmed in a study by Zhang et al. [70], who found that FUC suppressed secondary brain injury, cerebral edema, neurological deficits, and apoptosis in a mouse traumatic brain injury (TBI) model. Moreover, in primary neurons, FUC promoted neuronal survival and inhibited oxidative stress by activating Nrf/ARE signaling, while in Nrf^−^/^−^ knockout mice, the neuroprotective effect of FUC was abolished, and activation of autophagy was observed [70]. Furthermore, in vitro studies demonstrated that FUC attenuated LPS-induced neuroinflammation by decreasing secretion of inflammatory mediators, including tumor necrosis factor α (TNF-α), NO, interleukin (IL) 1β, IL-6, and prostaglandin E2 (PGE2) [90]. Suppression of MAPK/AP-1 and Akt/NF-κB pathways, reduction of expression of inducible nitric oxide synthase (iNOS) and cyclooxygenase 2 (COX2) are also involved in the anti-inflammatory activity of FUC [90]. Moreover, FUC was also observed to suppress neuroinflammation by affecting NLRP3 inflammasome by inhibiting C-CASP 1. Expression and oligomerization of that apoptosis-associated speck-like protein containing a C-terminal caspase recruitment domain (ASC) are the major components of the inflammasome. Interestingly, it was proposed that FUC may also modulate the initiation step of the inflammasome signaling pathways as FUC was noted to reduce pro-IL-1β and phosphorylated IκBα expression [81]. Lin et al. [91] showed that FUC attenuates cognitive impairment induced by scopolamine in a mouse Alzheimer’s Disease (AD) model. Treatment of mice with scopolamine increased acetylcholinesterase (AChE) activity, as well as decreased BDNF expression and choline acetyltransferase activity, which was reversed by FUC. Moreover, it was shown that FUC directly inhibited AChE (IC_50_ 81.2 µM) in a non-competitive manner, and based on molecular docking. It was found that FUC interacted with the peripheral anionic site of AChE [91]. A growing body of data from animal studies demonstrates the enormous potential of FUC in preventing disease or managing human health. Nevertheless, despite significant progress in characterizing its potential health-promoting effects, much research is still required to establish the appropriate protocol for human administration.

### 3.2. Astaxanthin

AST (3,3’-dihydroxy-β, β-carotene-4,4’-dione) is a natural xanthophylls [92,93]. Its predominant source in the diet are fish and seafood, mainly crawfish, crabs, shrimps, salmon, and pink trout [93,94,95]. The natural sources of AST are the algae *Haematoccocus pluvalis* [96,97], *Chlorella zofingensis*, as well as the yeast *Xanthophyllomyces dendrorhous* [77,95,96]. Eight isoprene units containing 40 carbon atoms form the compound’s chemical structure. The polyene carbon chain is terminated at both ends with β-ionone rings, each of which has one ketone group and one hydroxyl group in its structure. These groups are responsible for AST’s greater stability and polarity in relation to other known carotenoids [93]. It was proven that carotenoids containing a greater number of oxygen molecules with the same number of double bonds are characterized by higher photostability and more tremendous antioxidant potential [98]. The chemical structure of AST is responsible for its physicochemical and health-promoting properties. The hydrophobic carbon chain containing 9 conjugated double bonds and 2 unconjugated β-ionon rings is accountable for the quenching of ROS. The presence of oxygen in the end rings influences the hydrophilic properties of astaxanthin, contributing to the neutralization of free radicals and other oxidative substances in the aquatic environment. Such a hydrophilic-hydrophobic-hydrophilic structure of AST is analogous to the cell membrane, which allows it to be distributed across its entire width, enabling the removal of free radicals and ROS both on the surface of the membrane and inside it [92,93]. AST is characterized by a greater resistance to light and high temperature compared to other carotenoids and a free, rapid crossing of the BBB in animals [97].

The promising pro-health actions of AST include antioxidant, anti-inflammatory, antitumor, hepato-, cardio, and neuroprotective effects [77,78,79,93,94,95,96,97,98]. The potent antioxidant effect of AST manifests in high oxidative potential, production of chelate complexes with metals, and in the presence of metal ions generation of neutral radicals and aggregation into ester forms [99]. Wu et al. [100] assessed the effect of AST (intragastric administration; at the dose of 0.02% of daily diet 3 times a week) on D-galactose-induced brain aging in rats. It was observed that AST suppressed oxidative stress by enhancing the activity of antioxidant enzymes, including superoxide dismutase (SOD) and glutathione peroxidase, enhancing total antioxidant capacity and thiol levels. In addition, a reduction in the levels of antioxidant damage markers such as malondialdehyde (MDA), 8-hydroxy-2-deoxyguanosine, and protein carbonyl groups was observed in the brains of rats. Furthermore, AST augmented an anti-apoptotic index—Bcl2/Bax ratio as well as suppressed neuroinflammation as expressed by decreased COX2 expression. Moreover, it was found that the neuroregenerative properties of AST were associated with a reduction in histopathological changes in the hippocampus and an increase in BDNF expression in both the hippocampus and the brain of aging rats [100]. In a recent study, Aslankoc et al. [101] found that AST (oral administration, 100 mg/kg for 7 days) was protective against methotrexate damage in the hippocampus, cerebral cortex, cerebellar cortex, and blood in rats. In the control group, there was increased oxidative stress in the hippocampus, cerebral cortex, and blood, manifested by an increase in the total oxidative state (TOS) and a decrease in total antioxidant status (TAS), in contrast to the study group, in which AST alleviated oxidative stress. Moreover, AST suppressed histopathological changes in the hippocampus, cerebral cortex, and cerebellar cortex, including congestion, edema, and degenerative changes noted in the controls. In addition, AST was demonstrated to have anti-apoptotic and anti-inflammatory properties related to increased expression of myelin basic protein (MBP) and decreased CASP 3 levels, growth related oncogene (GRO), granulocyte colony-stimulating factor (GCSF), and iNOS [101]. Zhao et al. [102] suggested that AST could be a potential therapeutic agent for the treatment of neuropathic pain. C57BL/6 mice with spinal nerve ligation were administered intraperitoneally with AST at a dose of 5 mg/kg or 10 mg/kg from the 5th postoperative day for 23 days. It was shown that AST partially relieved neuropathic pain, and the analgesic effect was demonstrated on day 7. Subsequently, an in vitro study (spinal dorsal horns taken 11 days after spinal nerve ligation) found that AST reduced microglia activation and the expression of proinflammatory cytokines leading to inhibition of neuroinflammation. The anti-inflammatory effect of AST was manifested by the inhibition of p38 and Erk1/2 phosphorylation, as well as NFκB p65 nuclear translocation [102].

The promising use of AST in enhancing cognition is still being intensively researched. AST (administered orally at a dose of 25 mg/kg 5 times a week for 25 days) was shown to ameliorate memory impairment induced by doxorubicin in rats. Moreover, AST restored doxorubicin-induced histological changes in brain tissue, including degeneration and nuclear pyknosis in fascia dentata, hilus, and subiculum of the hippocampus, as well as focal hemorrhage in the area that separates the hippocampus from the striatum. Moreover, it was observed that the neuroprotective effect of AST is associated with a decrease in AChE activation, inhibition of oxidative stress, and overactive apoptotic processes [103]. In turn, Zhu et al. [104] assessed the effects of AST on cognition, oxidative stress, and neuroinflammation in mice with vascular dementia. AST (at a dose of 50 mg/kg, 100 mg/kg 200 mg/kg for 30 days) was shown to attenuate cognitive deficits in a dose-dependent manner as well as reduce oxidative stress, as observed by increasing SOD activity and reducing MDA. Moreover, a decrease in IL-1β expression and an enhancement of IL-4 expression were noted in the study group [104]. A recent study by Loganathan et al. [105] demonstrated that the astaxanthin-s-allylcysteine (AST-SAC) diester has a neuroprotective effect in alleviating cognitive deficits in diabetic rats by preventing spatial memory loss, as well as reducing brain tissue damage by inhibiting AChE activity, mitochondrial dysfunction, and oxidative stress. Moreover, in an in vitro study (SH-SY5Y neuronal cells treated with high glucose concentration), AST (at a dose of 5 μM, 10 μM and 15 μM) in a dose-dependent manner promoted neuronal viability by reducing the expression of pro-apoptotic proteins, leading to inhibition of apoptosis, increasing the level of endogenous antioxidant compounds reducing ROS generation, and by preventing mitochondrial dysfunction. The mechanism of preventing mitochondrial dysfunction goes on through modulating the membrane potential and the activity of oxidative phosphorylation complexes [105]. A randomized clinical trial, which enrolled 96 people (age 45–65) with mild memory impairment, investigated the effect of the AST-rich *Haematococcus pluvialis* extract. It was shown that in the group receiving AST at a dose of 6 mg/day and 12 mg/day for 12 weeks, improvement in performed tasks was significantly faster. That improvement was manifested by increased psychomotor speed, which is a marker of physical and mental coordination. Importantly, this study did not report any side effects from AST [106].

In addition to the research concerning the neuroprotective properties of AST alone in the treatment of CNS diseases, combination therapies are currently under consideration. Ata Yaseen Abdulqader et al. [107] assessed the effect of using AST in combination with valproic acid (VPA) in rats with pentylenetetrazole-induced epilepsy. VPA was shown to counteract the histopathological damage and behavioral disturbances induced by pentylenetetrazole. In contrast, AST alone (oral administration at a dose of 100 mg/kg) showed antiepileptic properties and increased anti-inflammatory activity compared to VPA alone. On the other hand, the use of AST/VPA combination therapy intensified the anti-epileptic effect, which was noted on the basis of the reduction of oxidative stress, the level of glutathione and TNF-α. Importantly, AST/VPA augmented the improvement in animal behavioral changes compared to VPA alone [107].

### 3.3. Siphonaxanthin

Siphonaxanthin (3,3′,19-trihydroxy-7,8-dihydro-8-oxo-a-carotene), a keto-carotenoid present in edible green algae including *Codium fragile*, *Caulerpa lentillifera*, *and Umbraulva japonica*, constitutes approximately 0.03–0.1% of their dry weight [108]. Siphonaxanthin contains an additional hydroxyl group that could contribute to its strong apoptosis-inducing effect [109]. The biological functions of this ketocarotenoid are associated with antioxidant activity [110], anti-inflammatory effect [111], suppression of cell viability, induction of apoptosis, more potent anti-angiogenic activity than FUC [112,113,114], and antiobesity [115,116]. However, additional in vivo studies, are needed to validate siphonaxanthin’s bioavailability and biological action.

Dambeck and Sandmann [110] showed that siphonaxanthin exerts an efficient outcome against the radical formation and lipid peroxidation [110]. Studies performed on the transfected human monocytic cell line, which was treated with siphonaxanthin at 1.0 µM concentration for 24 h, showed the anti-inflammatory effect of this carotenoid, manifested by the significant inhibition of the LPS- and TNF-α-induced NFκB activation. Moreover, pretreatment with siphonaxanthin at 1.0 µM concentration significantly suppressed the IL-1β-induced NFκB activation [117]. Ganesan et al. [113] revealed that siphonaxanthin might inhibit FGF-2 signaling. Human endothelial cells treated with siphonaxanthin at 0.1 and 0.5 µM concentration for 6 h exhibited the inhibition of FGF-2-induced intracellular proliferation and survival signals by down-regulating the FGF-2-induced phosphorylation of Akt and the Erk1/2 [113]. FGF signaling often leads to the concurrent activation of both the Raf/MAPK and the PI3K/Akt pathways, which, as was mentioned, are crucial for neurons survival or synaptic plasticity [118]. In addition, this carotenoid, at a concentration of 20 μM, reduced human leukemia cell viability (*p* < 0.05) within 6 h of treatment, inducing the apoptosis by decreasing expression of Bcl-2 and increasing activation of CASP 3 [112].

### 3.4. Mytiloxanthin

Mytiloxanthin (3,3′,8′-trihydroxy-7,8-didehydro-β,κ-caroten-6′-one), a metabolite of FUC, is another carotenoid with high antioxidant properties. Mytiloxanthin is widely distributed in marine mussels, oysters, and tunicates [77,119]. As it was mentioned before, carotenoids singlet oxygen-quenching activity depends on the number of conjugated double bonds, polyene chain structures, and functional groups, therefore Mytiloxanthin, which has more conjugated double bond than FUC, is suggested to have stronger quenching activity for singlet oxygen.

Maoka et al. [120] investigated the anti-oxidative activities of mytiloxanthin, they revealed that it exhibits a high quenching activity of singlet oxygen (61.6%) similar to that of AST (61.0%). Moreover, the researchers showed this compound has more powerful inhibitory activity on lipid peroxidation (20% formation of lipid hydroperoxide) than AST (24%), FUC (32%), and β-carotene (38%), at a final concentration of 167 μM [120].

### 3.5. Saproxanthin and Myxol

The two monocyclic carotenoids, seldom found in nature, (3R)-Saproxanthin and (3R,2′S)-Myxol, are produced by *Saprospira grandis*, marine bacterial strain 04OKA-13-27 and *Anabaena variabilis* ATCC 29413, marine bacterial strain P99-3, YM6-073, respectively [121]. These tetraterpenes are reported to possess neuroprotection against L-glutamate toxicity, lipid peroxidation prevention, and have powerful antioxidant potential, for instance, by their orientation in the head-group region of the phospholipids that form the bilayer, which leads to the reinforcement and stabilization of biological membranes. Therefore, these carotenoids may induce a reduction of membrane permeability to oxygen and may enhance protection against radical-induced peroxidation [122,123].

As was revealed by Shindo et al. [121] the antioxidant activity of saproxanthin and myxol is stronger than that of β-carotene or zeaxanthin. The team used the rat brain homogenate model to evaluate the inhibitory activities against lipid peroxidation by saproxanthin, myxol, and zeaxanhin. Saproxanthin showed the most potent effect with IC_50_ value 2.1, whereas myxol and zeaxanhin were 6.2 and 13.5 μM, respectively. Furthermore, the researchers tested saproxanthin and myxol inhibitory activities against L-glutamate toxicity in embryonic rat retinal neuron hybrid cells, and their concentration necessary to reduce glutamate-induced cell death by 50% (EC_50_ values) was 3.1 and 8.1 μM, respectively. At the same time the protective effect of AST and β-carotene was >500 and >100 μM, respectively [121]. The results show that these rare carotenoids might be expected to be useful for ameliorating tissue damage resulting from free radicals’ generation and subsequent cell membrane peroxidative deterioration, as well as possessing potent neuroprotective effect against L-glutamate toxicity and they may be helpful in cerebral ischemic disease treatment.

The biological effect of marine carotenoids described above are summarized in the Table 1 and Table 2.

## 4. Conclusions

There is considerable scientific and social interest in the use of natural compounds in the prevention and treatment of many diseases, including neurological disorders. In recent years, the demand for biologically active nutraceuticals implies the search and development of natural sources of these molecules. This led to an interest in marine compounds as unused and new natural sources. Marine sources are a great wealth of bioactive substances that have a beneficial effect on the human body, including carotenoids, which has been indicated in many scientific reports. There is strong scientific evidence that the marine carotenoids fucoxanthin and astaxanthin support the CNS. In turn, noteworthy as raising high hopes are mytiloxanthin, saproxanthin, and myxol. The enhancement of neuroplasticity by marine carotenoids is mainly related to anti-inflammatory and antioxidant effects. Nevertheless, activation of pathways related to neurogenesis and synaptogenesis mean that these compounds appear to have great therapeutic potential. However, it is necessary to conduct further preclinical and clinical studies that will be able to accurately determine the mechanism of their action, as well as dosing in particular CNS diseases.

## Figures and Tables

**Table 1:** In vitro studies of biological roles of marine carotenoids.

Carotenoid	Effect	Model	Bioactive Concentration	Target	Ref.
Fucoxanthin	neuroprotection	rat cortical neurons	5, 10 and 20 μM	Nrf2 signaling	[88]
	neuroprotection	PC12 cells	0.5, 1, 2 and 5 μM	Nrf2 signaling	[89]
	anti-neuroinflammation	BV-2 microglial cells	5, 10, and 20 μM	MAPKs and NF-κB signaling	[90]
	anti-neuroinflammation	bone marrow-derived macrophages, bone marrow-derived dendritic cells, astrocytes	40 μM	NF-κB and NLRP3 inflammasome signaling	[81]
Astaxanthin	anti-neuroinflammation	BV2 cells, PC12 cells, primary astrocytes	5 or 10 μM	MAPKs and NF-κB signaling	[102]
	neuronal viability	human neuronal cell line SH-SY5Y	5, 10 and 15 μM	pro-apoptotic proteins	[105]
Siphonaxanthin	anti-neuroinflammation	human monocytic cells	1 μM for 24 h	NF-κB signaling	[117]
	neuron survival synaptic plasticity	human endothelial cells	0.1 and 0.5 μM for 6 h	FGF-2 signaling	[113]
	anti-proliferative	human leukemia cells	20 μM	Bcl-2, CASP 3	[112]
Saproxanthin and Myxol	neuroprotection	embryonic rat retinal neuron hybrid cells	3.1 and 8.1 μM, respectively	L-glutamate toxicity	[121]

**Table 2:** In vivo and clinical studies of biological roles of marine carotenoids.

Carotenoid	Effect	Model	Bioactive Concentration	Target	Ref.
Fucoxanthin	neuroprotection	rat stroke	30, 60 and 90 mg/kg	Nrf2 signaling	[88]
	neuroprotection	zebrafish	6.25, 12.5, 25 and 50 μg/mL	Nrf2 signaling	[89]
	neuroprotection	traumatic brain injury mice	50, 100 and 200 mg/kg	Nrf2/ARE signaling	[70]
	cognitive impairments attenuation	Alzheimer’s Disease mice	50, 100 and 200 mg/kg	AChE, BDNF	[91]
Astaxanthin	antioxidation anti-neuroinflammation, neuroregeneration	rats’ brain	0.02% of daily diet, 3 times a week	antioxidant enzymes COX2, BDNF	[100]
	anti-apoptotic, anti-inflammation, oxidative stress alleviation	rats	100 mg/kg for 7 days	MBP, CASP 3, iNOS	[101]
	neuropathic pain alleviation	C57BL/6 mice	5 or 10 mg/kg for 23 days	MAPKs and NF-κB signaling	[102]
	neuroprotection	rats	25 mg/kg 5 times a week for 25 days	AChE	[103]
	oxidative stress alleviation	vascular dementia mice	50, 100 and 200 mg/kg for 30 days	SOD, MDA, IL-4, IL-1β	[104]
	psychomotor speed improvement	people with mild memory impairment	6 and 12 mg/day for 12 weeks	–	[106]
	Antiepileptic anti-inflammation	epileptic rats	100 mg/kg	–	[107]

## Data Availability

No new data were created or analyzed in this study. Data sharing is not applicable to this article.
